# Frequency of headache among the employees of a rubber company in the state of São Paulo, Brazil

**DOI:** 10.1590/S1516-31802011000200003

**Published:** 2011-03-03

**Authors:** Juliana Stuginski-Barbosa, José Geraldo Speciali

**Affiliations:** I MSc. Surgeon Dentist, Department of Neurosciences, Faculdade de Medicina de Ribeirão Preto, Universidade de São Paulo (FMRP-USP), Ribeirão Preto, São Paulo, Brazil.; II MD. Associate Professor of Neurology, Department of Neurosciences, Faculdade de Medicina de Ribeirão Preto, Universidade de São Paulo (FMRP-USP), Ribeirão Preto, São Paulo, Brazil.

**Keywords:** Occupational health, Headache, Migraine disorders, Tension-type headache, Absenteeism, Saúde do trabalhador, Cefaleia, Transtornos de enxaqueca, Cefaleia tipo tensional, Absenteísmo.

## Abstract

**CONTEXT AND OBJECTIVE:**

Primary headaches may be responsible for absenteeism and a fall in the yield and productivity of work. The aim of this study was to establish the presence and frequency of primary headache among employees of a rubber shoe sole company, and its link to absenteism.

**DESIGN AND SETTING:**

Cross-sectional study carried out with help from the staff of the medical and social department of a rubber factory located in the municipality of Franca, São Paulo.

**METHOD:**

A questionnaire on headache characteristics was distributed to all employees. The returned and completed questionnaires were divided into two groups: with and without reports of headache. The headaches were classified into four main groups: migraine, tension-type headache (TTH), cluster headache and others. In terms of the reported frequency, headaches were also classified as chronic daily headache (CDH).

**RESULTS:**

The number of valid questionnaires was 392 (59%); 80.9% were from male and 19.1% from female employees. Headaches were reported by 120 subjects (30.6%), with 17.4% belonging to the migraine group and 8.9% to the TTH group. Migraine was more frequent (p < 0.001) among all participants and also among the women (p < 0.05). TTH was more frequent among the men (p < 0.05). CDH was identified in 14 individuals (3.6%).

**CONCLUSIONS:**

Headache was a common problem among the employees of this company and was a cause of absenteeism for 8.7% of the respondents to the questionnaire.

## Abstract (pt)

**CONTEXTO E OBJETIVO:**

Cefaleias primárias podem ser causa de absenteísmo, e de queda no rendimento e na produtividade do trabalho. O objetivo do estudo foi estabelecer a presença e frequência de cefaleia primária em funcionários de uma empresa de solados de borracha,e sua relação com absenteísmo.

**TIPO DE ESTUDO E LOCAL:**

Estudo transversal realizado com a ajuda do pessoal do departamento médico e social de uma empresa localizada no município de Franca, São Paulo.

**MÉTODO:**

Um questionário sobre as características da cefaleia foi distribuído a todos os funcionários. Os questionários devolvidos e preenchidos foram divididos em dois grupos: com e sem relato de cefaleia. As cefaleias foram classificadas em quatro grupos principais: migrânea, cefaleia do tipo tensional (CTT), cefaleia em salvas e outras cefaleias. Através da frequência da cefaleia, foi possível a classificação em cefaleia crônica diária (CCD).

**RESULTADOS:**

O número de questionários válidos foi de 392 (59%), 80,9% questionários eram de funcionários do gênero masculino, e 19,1%, do gênero feminino. Cento e vinte (30,6%) dos funcionários relataram apresentar cefaleia, sendo que 17,4% pertenceram ao grupo migrânea e 8,93% ao grupo CTT. O diagnóstico de migrânea foi o mais frequente (p < 0,001) dentre todos, e também entre as mulheres (p < 0,05). O diagnóstico de CTT foi mais frequente entre os homens (p < 0,05). CCD foi identificada em 14 indivíduos (3,6%).

**CONCLUSÕES:**

Cefaleia é um problema frequente entre os funcionários da empresa participante da pesquisa sendo a causa de absentismo em 8,7% dos que responderam o questionário.

## INTRODUCTION

Epidemiological studies usually start from clinical observations that generate data and allow hypotheses to be established.^1^ Population-based epidemiological studies indicate that the prevalence of chronic pain ranges from 19% to 46% depending on the population, and the age and occupation of the subjects.^2,3^ The prevalence of chronic pain among the employees of a Brazilian university was 61.4%, and the head was the most prevalent site of pain (26.7%).^4^ High prevalence of pain, including primary headaches, may contribute towards greater demand for treatment in neurologists' offices.

In view of the impact of pain and its association with depression, anxiety and even mortality, identification of risk groups presenting pain as a symptom is extremely important.^5^

The combination of clinical observations and epidemiological investigations is particularly important in studies relating to headache. Patients who seek treatment at specialized centers are systematically different from individuals with headache in the general population, who often do not receive a diagnosis or treatment and therefore are not the target of clinical observations.^1^

In a study conducted in the United States, 30,000 workers were interviewed about the impact of health on work. In that study, the total cost of health problems was estimated as 250 billion dollars per year, and 60 billion dollars was attributed to problems relating to pain. Among these, headache produced a cost of about 20 billion dollars in terms of loss of productivity.^6^

Primary headaches may be responsible for absenteeism, a fall in the yield and productivity of work or study and loss of leisure days, as well as affective and relationship problems.^7–9^

Primary headache is considered to be pain that occurs in the head without a temporal relationship with another disorder that might be recognized as a cause of headache. The most prevalent primary headaches are migraine and tension-type headache (TTH), which are classified in groups 1 and 2 of the International Headache Classification.^10^

## OBJECTIVE

The objective of the present study was to observe the frequency of primary headaches, especially migraine and TTH, among the employees of a rubber shoe sole factory located in the interior of the state of São Paulo, and the absenteeism linked to it.

## METHODS

This cross-sectional study was carried out with help from the staff of the medical and social department of a company located in the municipality of Franca, São Paulo, which manufactures rubber products and is currently considered to be one of the most important industrial complexes in the footwear sector (Componam Componentes para Calçados Ltda).

A self-administered questionnaire (Annex 1) drawn up by professionals at the Headache and Craniofacial Pain Outpatient Clinic of the University Hospital, Faculdade de Medicina de Ribeirão Preto, Universidade de São Paulo (HCFMRP-USP),^11^ was distributed to all the employees, who were instructed to fill it out at home. The questionnaire contained items regarding demographic data and 24 questions about the topic under investigation.

The company had 666 production employees over the age of 18 years who were currently working, among whom the questionnaires were distributed. These workers were organized into four different shifts. The minimum sample size was calculated assuming a standard error of 5%, 95% confident interval and estimated response rate of 50%. This resulted in a minimum sample size of 244 participants.

To avoid embarrassment and maintain anonymity, study participants were not required to state their names on the questionnaire, although they could so optionally. One question was open and the remaining ones were multiple choice questions. The question "do you habitually have headaches?" was defined in order to assign the employees to groups with or without headache. If an employee reported the presence of headache, he was then asked to answer questions regarding the characteristics of the disorder such as frequency, duration, location, intensity and related symptoms. The questions were drawn up in such a way as to enable classification of headaches in accordance with the criteria of the International Classification of Headache Disorders (Headache Classification Subcommittee of the International Headache Society).^10,11^ A question about absenteeism was added to the questionnaire. The question about headache intensity required choosing a value between 0 (absence of pain) and 10 (the strongest pain the person could feel).

After filling out the questionnaire, the employees deposited it in a box located in the company's social department. Returning the questionnaire was optional and was supposed to occur within 15 days.

The study was approved by the Ethics Committee of Santa Casa de Franca under no. 022–2008 and complied with the standards and regulations for research involving human beings issued by the National Health Board (Conselho Nacional de Saúde; CNS) under its Resolution 196/96. A consent form was delivered to each participant for signing together with the questionnaire. A telephone number and an electronic address were available so that the employees could contact the investigators if they had any queries. The employees also had the possibility of clarifying their queries through the company's medical and social department.

The questionnaires that were filled out and returned were divided into two groups: those that reported habitual headaches and those that did not. On the basis of the characteristics reported by the employees, it was possible to classify the headaches into four major groups: migraine, tension-type headache (TTH), cluster headache and other headaches. Questionnaires containing data that were not enough to characterize the type of headache were assigned to a fifth group named insufficient data.

For migraine, the required characteristics were: headache attacks lasting 4–72 hours (untreated or unsuccessfully treated); presence of at least two of the following characteristics: unilateral location, pulsating quality, moderate or severe pain intensity, aggravation by or causing avoidance of routine physical activities; and, during the headache, at least one of the following: nausea and/or vomiting, photophobia and phonophobia. For TTH, the required characteristics were: headache lasting from 30 minutes to 7 days; presence of at least two of the following characteristics: bilateral location, pressing/tightening (non-pulsating) quality, mild or moderate intensity without aggravation by routine physical activity such as walking or climbing stairs; and no nausea or vomiting (anorexia may occur) and no more than one occurrence of photophobia or phonophobia. For cluster headache, the required characteristics were: severe or very severe unilateral orbital, supraorbital and/or temporal pain lasting 15–180 minutes if untreated, accompanied by at least one of the following: ipsilateral conjunctival injection and/or lacrimation, ipsilateral nasal congestion and/or rhinorrhea, ipsilateral eyelid edema, ipsilateral forehead and facial sweating, ipsilateral miosis and/or ptosis, and a sense of restlessness or agitation.

On the basis of the frequency reported, it was also possible to determine the number of employees who had chronic daily headache (CDH), and the groups were also subdivided into episodic migraine, chronic migraine, episodic TTH and chronic TTH.

The variables regarding the demographic data and smoking habit were compared between the groups.

The Shapiro-Wilk test was applied to determine the distribution of the age variable. The Mann-Whitney test was used to compare ages between the groups and the t-test for two independent samples was used to compare the intensity of headache between the groups. The chi-square test or Fisher exact test was applied to analyze categorical variables according to the frequency expected in the cells. The hypothesis test for two proportions was applied to compare the data with those reported in the literature.

## RESULTS

A total of 666 questionnaires were distributed and 393 of them (59%) were returned. One questionnaire was returned without being filled out and 392 were valid; 317 (80.87%) were returned by male employees and 75 (19.13%) by female employees.

The overall mean age was 35.4 years, the male mean age was 36.6 years and the female mean age was 30.5 years. The male mean age was significantly greater than the female mean age (p < 0.001); 10.5% of the participants did not state their age.

Only 4.2% of the individuals (16) did not answer the questions regarding headache correctly and were therefore not assigned to any of the headache groups. The remaining subjects were divided into two groups, i.e. subjects who reported habitual headaches and subjects who did not. There were 120 employees (30.61%) with reports of suffering from headache. Table 1 lists demographic information (gender, schooling, marital status and age range) and smoking habit data for both groups. The mean age of the subjects with headaches was 33.5 years and the mean age of the subjects without headaches was 36.3 years, with no significant difference between the groups (p > 0.05). Seventy-eight men (24.6%) and 42 women (56%) reported suffering from headaches. The proportion of women with headache was significantly greater than the proportion of men (p < 0.001). There was no significant difference in schooling, marital status, age range or smoking habit between subjects with and without headaches (p > 0.10).

**Table 1.** Sample characteristics regarding the presence of headache

		Headache		Total – n (%)
		Yes – n (%)	No – n (%)	
Gender	Male	78 (24.6)*	239 (75.4)	317 (100)
	Female	42 (56)*	33 (44)	75 (100)
**Total**		**120 (30.6)**	**272 (69.4)**	**392 (100)**
Schooling	Not declared	7 (46.7)	8 (53.3)	15 (100)
	Up to 4^th^ grade	2 (11.1)	16 (88.9)	18 (100)
	Elementary school completed	17 (32)	36 (68)	53 (100)
	High school completed	67 (32.2)	141 (67.8)	208 (100)
	Technical course	6 (21.4)	22 (78.6)	28 (100)
	Higher education	21 (30)	49 (70)	70 (100)
**Total**		**120 (30.6)**	**272 (69.4)**	**392 (100)**
Marital status	ND	10 (32.2)	21 (67.8)	31 (100)
	Married	73 (29.5)	174 (70.5)	247 (100)
	Single	32 (31)	71 (69)	103 (100)
	Divorced	3 (33.3)	6 (66.7)	9 (100)
	Widowed	2 (100)	0 (0)	2 (100)
**Total**		**120 (30.6)**	**272 (69.4)**	**392 (100)**
Age range	18 | −20	2 (33.3)	4 (66.7)	6 (100)
	20 | −30	24 (32.4)	50 (67.6)	74 (100)
	30 | −40	37 (35)	69 (65)	106 (100)
	40 | −50	28 (28.5)	70 (71.5)	98 (100)
	50 | −60	15 (25)	45 (75)	60 (100)
	≥ 60	1 (14)	6 (86)	7 (100)
	Age not mentioned	13 (31.7)	28 (68.3)	41 (100)
**Total**		**120 (30.6)**	**272 (69.4)**	**392 (100)**
Smoker	Yes	9 (25.7)	26 (74.3)	35 (100)
	No	111 (31)	246 (69)	357 (100)
**Total**		**120 (30.6)**	**272 (69.4)**	**392 (100)**
**Total**		**120 (30.6)**	**272 (69.4)**	**392 (100)**

* significant difference (chi-square test, P < 0.001); no significant difference (chi-square test, P > 0.05).

Table 2 presents the distribution of the subjects (divided according to gender) who reported headaches in accordance with the headache diagnoses of the International Headache Classification. Among the employees who answered the questionnaire (n = 392), 17.3% belonged to the migraine group and 8.9% to the TTH group. The diagnosis of migraine was significantly more frequent (p < 0.001) and was proportionally more frequent among the women (p < 0.05), whereas the diagnosis of TTH was more frequent among the men (p < 0.05).

**Table 2.** Groups of headache diagnoses by gender, in accordance with the International Classification of Headaches (2004), in the total sample (n = 392)

	Gender		Total
	Male	Female	
Headache subtype			
Migraine*	37 (9.5%)	31 (7.9%)†	68 (17.4%)
Tension-type headache	28 (7.2%)‡	7 (1.8%)	35 (8.9%)
Cluster headache	0 (0.0%)	1 (0.2%)	1 (0.2%)
Other headaches	9 (2.3%)	2 (0.5%)	11 (2.8%)
Insufficient data	4 (1.0%)	1 (0.2%)	5 (1.2%)
**Total**	**78 (20%)**	**42 (10.6%)**	**120 (30.6%)**

* significantly more frequent (chi-square test, P < 0.001);

† significantly more frequent in women than in men (chi-square test, P < 0.05);

‡ significantly more frequent in men than in women.

Table 3 presents the data regarding the frequency of headache according to diagnostic group. Headaches occurred frequently, on one to seven days per month in 40% of the subjects who reported it. CDH was identified in 14 individuals (3.57%), chronic migraine in 12 (3.06%) and chronic TTH in two (0.51%). Chronic migraine was proportionally and significantly more frequent than chronic TTH (p < 0.05).

**Table 3.** Frequency of each headache subtype classified in accordance with the criteria of the International Classification of Headaches (IHS-II), among patients with headache (n = 120)

Frequency	Migraine*	Tension-type headache*	Cluster headache	Other headaches	Insufficient data	Total
Every day of the month	3	0	0	0	0	3 (2.5%)
> 15 days per month	9	2	0	0	0	11 (9.2%)
8–15 days per month	10	5	0	3	1	19 (15.8%)
1–7 days per month	28	17	0	2	1	48 (40%)
4–11 attacks per year	11	6	1	4	0	22 (18.3%)
< 4 attacks per year	7	5	0	2	0	14 (11.7%)
Not declared	0	0	0	0	3	3 (2.5%)
**Total**	**68**	**35**	**1**	**11**	**5**	**120 (100%)**

* Chronic migraine (more than 15 days per month) was proportionally and significantly more frequent than chronic tension-type headache (chi-square test, P < 0.05).

**Figure 1.**
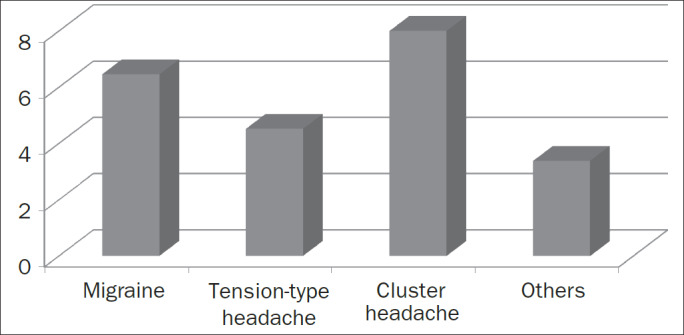
Headache intensity by subgroup according to the International Classification of Headache (IHS-II, 2004).

All the subjects assigned values to the intensity of headache, with a mean of 5.94 and a median of 6. Figure 1 illustrates the intensity of pain in the different headache groups. Subjects in the migraine group had significantly greater headache intensity than did subjects with TTH (p < 0.001).

Ten (8.3%) of the 120 subjects in the headache group (six women and four men) reported having missed work because of the condition. Nine of these belonged to the migraine group and one to the cluster headache group.

## DISCUSSION

Among the employees, 30.61% had headaches, and the most frequent forms were migraine and TTH. About 17% of the workers studied (n = 392) were assigned to the migraine group. This result was similar to that reported by Pop et al. in a study conducted in a Dutch factory in which 15% of the employees had migraine.^12^

Migraine is a recurrent primary headache that manifests as crises lasting for four to 72 hours, preferentially of unilateral localization. It has a pulsatile nature, with moderate or strong intensity exacerbated by physical activity and associated with nausea and/or vomiting or photophobia or phonophobia. It is divided into two main subtypes: migraine with aura and migraine without aura.^10^

TTH is the most common type of primary headache and, at the same time, the least studied. TTH is divided into two groups: episodic (ETTH) and chronic (CTTH). In ETTH, the pain typically occurs as a sensation of weight or pressure of weak to moderate intensity and has a bilateral frontotemporal or occipital location. It does not become worse with physical activity and is not accompanied by nausea, but may involve photophobia or phonophobia. CTTH has the same characteristics as ETTH, but occurs on more than 15 days per month and lasts for more than three months, affecting patients with a history of ETTH.^10^

Migraine and TTH are also the most prevalent types in population studies. In a study in the city of Ribeirão Preto, the prevalence of primary headaches was found to be 49.9%, with predominance in the female gender (63.8%). The most prevalent diagnoses were ETTH (16.2%), migraine without aura (13.9%) and migraine with aura (5.1%). Both migraine and ETTH were more prevalent among women, with migraine affecting 28% of women and 9.9% of men, and ETTH affecting 23.2% of women and 9.5% of men.^11^

Because patients with TTH present less intense headaches with a smaller individual impact, they tend to neglect their pain,^13^ and this may have contributed towards failure to respond to the questionnaire in the present study. It may also explain the different percentages of TTH that have been found in different populations. In the present study, migraine was significantly more frequent among women and TTH was more frequent among men.

Another type of primary headache that can be characterized in a questionnaire is cluster headache. This type of headache involves strictly unilateral strong pain crises in the orbital, supraorbital and/or temporal region that last for 15 to 180 minutes. The crises are accompanied by one or more autonomic symptoms ipsilaterally to the pain, such as conjunctival hyperemia, tearing, nasal congestion, rhinorrhea, sudoresis on the forehead and face, miosis, ptosis and palpebral edema. During the crises, most patients are restless or agitated.^10^ Cluster headache is uncommon in comparison with migraine, and its population-based prevalence is lower than 1%, with predominance among males.^14^ In the present study, the single subject assigned to the cluster headache group was a female.

Regarding the frequency of headache crises, 40% of the subjects (41.2% belonging to the migraine group and 48.6% belonging to the TTH group) reported that they suffered headaches on one to seven days per month. This result is similar to the findings of Bigal et al., who observed a crisis frequency of two to four times a month in 47% of the employees with migraine.^15^

Very frequent crises (occurring on more than 15 days per month) characterize a group of headaches called chronic daily headache (CDH).^16^ The presence of CDH affects the quality of life, with an important impact on individuals' productive, social and emotional characteristics.^17^ The prevalence of CDH in the population ranges from 4.1 to 6.4%.^18,19^ In the study conducted on the population of Ribeirão Preto, 2.6% of the individuals presented CDH: 2% with chronic migraine and 0.6% with chronic TTH.^11^ In the present study, 14 subjects (3.57%) fulfilled the criteria for CDH, with no significant difference compared with the proportions reported in the literature (P > 0.05). Based on clinical observations, chronic migraine and TTH are believed to progress gradually from their episodic to their chronic forms.^20^ Among these 14 individuals (3.57%), 12 (3.06%) fulfilled the criteria for chronic migraine, and this result was similar to what was reported in the study by Queiroz et al.^19^ Thus, chronic migraine was significantly more frequent than chronic TTH.

78.1% of the patients with primary headache report that headache interferes with their general living activities and 41.7% report interference with their quality of life.^11^

Individuals' ability to work is definitely affected by their health condition. Losses in productivity can be evaluated both in terms of absenteeism and presenteeism. Absenteeism refers to missed days of work, leaves of absence and work disability, while presenteeism refers to reduction of productivity due to disease by an employee who remains present on the job. Presenteeism is estimated to represent 86% of the loss of productivity and absenteeism to represent 16% of it.^21^

The presence of headache, especially migraine, can be considered to be a risk factor for loss of productivity on the job. The expenses relating to headache can be direct (expenses relating to the healthcare system) and indirect (losses due to missed days of work, reduced productivity and fewer opportunities for promotion and education).^22–24^

Among the patients with headache, migraineurs experience a high level of pain and disability. Less than 10% of them state that they are fit for work during the crises.^1^ The prevalence of migraine is higher between 22 and 66 years of age, thus coinciding with the time of peak productivity of the affected subjects. At 30 years of age, women tend to present higher intensity and frequency of crises, and this may increase the risk that migraine may interfere with productivity.^25,26^

A study conducted on workers in France reported that the presence of migraine resulted in a relative risk of 1.79 for the reduction of attention and a risk of 1.46 for the reduction of time at work.^27^ A study conducted at the University Hospital of Ribeirão Preto estimated a loss of approximately 500,000 dollars in a single year due to the reduced productivity of its employees during migraine crises.^15^

Even though impairment of work was reported, absenteeism was not frequent (8.7%) among the participants; 90% of those who reported missing work due to headache belonged to the migraine group. Headache also generated loss of productivity, even when the employees went to work with a headache. These occurrences cause losses both to the company and to the workers.

The instrument selected for data collection in the present study was a questionnaire. Such instruments have a good cost/benefit ratio and present certain advantages such as low cost and rapidity of use, provision of direct knowledge of the realities, absence of investigator's bias, and guaranteed participant anonymity. Asking the subjects to fill out the questionnaire individually at home may have been a favorable point, since it gave the employees more time and availability to respond.

However, the present instrument also had some limitations, and one of them was the large number of questions that were asked about the topic under investigation, which may have discouraged potential responders. The absence of the investigator while the questionnaire was being answered was minimized by the researchers' offer to clarify any queries relating to filling it out. The response rate was as expected but because it was not 100%, this may have compromised the representativeness of the data collected. Only one questionnaire was returned unanswered. The question with the largest number of missed replies (10.5%) was the one relating to the subject's age. However, this did not impair the results obtained.

Studies conducted in the United States have indicated that, even though epidemiological surveys have shown that the prevalence of migraine has stabilized over the years, the number of patients diagnosed has increased. This demonstrates the efforts that have been made by clinicians and neurologists towards diagnosing and treating this very common headache. However, despite these efforts, 50% of all migraineurs never receive a diagnosis. The first step needed to change this picture is to identify these individuals and encourage them to seek adequate treatment for their problem.^1^

Knowledge about the impact of the disease can help healthcare professionals to understand the severity of headaches and how the condition affects their patients' lives. Better communication between patients and health professionals regarding the disability relating to headaches can potentially improve these individuals' medical treatment. In turn, improved treatment can reduce emergency care, abuse of medication and loss of productivity.^9^

Implementation of educational programs for physicians and company employees in order to teach them about headaches may help to improve the understanding of this symptom.^12^ In one example, there was a significant improvement in quality of life, a reduction in the disability associated with headaches and stimulation of the use of self-care among the employees who participated in an educational program.^28^ Information about the employees' clinical situation made it possible for the company's medical and social department to plan preventive approaches directed towards the problems, as well as reducing their impact on work.

Investments by companies in educating their physicians and employees about the aspects of headache discussed here may transform the damage into gains. In addition, these employees' quality of life will improve, thereby transforming them into individuals who are more productive and better satisfied with the activities that they perform.

## CONCLUSION

Headache was a frequent problem among the employees of the company participating in the present study, and was a cause of absenteeism among 8.7% of those who answered the questionnaire.
